# Reality Check for the Chinese Microblog Space: A Random Sampling Approach

**DOI:** 10.1371/journal.pone.0058356

**Published:** 2013-03-08

**Authors:** King-wa Fu, Michael Chau

**Affiliations:** 1 Journalism and Media Studies Centre, The University of Hong Kong, Hong Kong, China; 2 School of Business, The University of Hong Kong, Hong Kong, China; Umeå University, Sweden

## Abstract

Chinese microblogs have drawn global attention to this online application’s potential impact on the country’s social and political environment. However, representative and reliable statistics on Chinese microbloggers are limited. Using a random sampling approach, this study collected Chinese microblog data from the service provider, analyzing the profile and the pattern of usage for 29,998 microblog accounts. From our analysis, 57.4% (95% CI 56.9%,58.0%) of the accounts’ timelines were empty. Among the 12,774 non-zero statuses samples, 86.9% (95% CI 86.2%,87.4%) did not make original post in a 7-day study period. By contrast, 0.51% (95% CI 0.4%,0.65%) wrote twenty or more original posts and 0.45% (95% CI 0.35%,0.60%) reposted more than 40 unique messages within the 7-day period. A small group of microbloggers created a majority of contents and drew other users’ attention. About 4.8% (95% CI 4.4%,5.2%) of the 12,774 users contributed more than 80% (95% CI,78.6%,80.3%) of the original posts and about 4.8% (95% CI 4.5%,5.2%) managed to create posts that were reposted or received comments at least once. Moreover, a regression analysis revealed that volume of followers is a key determinant of creating original microblog posts, reposting messages, being reposted, and receiving comments. Volume of friends is found to be linked only with the number of reposts. Gender differences and regional disparities in using microblogs in China are also observed.

## Introduction

The Internet market in China has been recognized as a rapidly expanding industry for more than a decade. In fact, it is still growing but its growth rate seems to have stabilized. According to statistics [1], the Chinese Internet population increased 56 million users in 2011 and the country’s Internet penetration rate rose only four percentage points in a year compared with six percentage points yearly growth rate since 2007. Currently, total Internet users in China have reached 538 million by the end of June, 2012, penetrating 39.9% of the country’s population. About 72.2% of Chinese Internet subscribers have obtained online access by hand-held devices. Moreover, Chinese people are spending longer hours online than before, such that average weekly time spent online per person elevated from 18.3 hours in 2010 to 19.9 hours in 2012 [1].

One significant shift in the pattern of Chinese people’s online use is the emergence of social media. In 2012, one of every two Chinese netizens used “Weibo” – the Twitter equivalent service in China, whereas traditional modes of online communication appear to be diminishing [1]. Use of email decreased from 54.6% in 2010 to 48.1% in 2012, and the adoption rate for online discussion forums declined from 32.4% in 2010 to 29.0% in 2012 [1].

Currently, the two leading microblog platforms in China are Sina Weibo and Tencent (QQ) Weibo. Sina Weibo (weibo.com) started its services in August 2009 and Tencent Weibo service (t.qq.com) was launched in April 2010. Both claimed to have more than 200 million registered accounts by the end of 2011 [2]. Other players include Sohu, Baidu, ifeng, and NetEase.

According to a market research study [3], 56% of Chinese microbloggers were male and 44% were female. Those under 30 years of age constituted approximately 70% of the Chinese microbloggers. They were mostly well educated – tertiary and university degree holders comprised more than 80% of the total microbloggers. Over 40% earned more than 2,000 RMB (approximately 317 U.S. dollars) per month, while the average per-capita wage of urban Chinese is roughly 1,284 RMB (203 U.S. dollars) per month [4].

Despite heavy-handed regulatory measures like content censorship, account suspension, and the implementation of a real identity registration system [5], Chinese microblogs have been frequently and vibrantly discussed with respect to their capacity to bring the country political or societal changes [6], [7], [8], [9], [10]. Using microblogs, Chinese netizens have broken a number of news reports about social injustice or conflict between local governments and peasants. Some well-known examples are “My Father is Li Gong” [11] and “Death of Qian Yuen Hui” [12]. Such citizen journalistic functions of microblogging constitute one of the key concerns of the scholars and the western media. Although this concern is prevalent in the mass media and political circles, mass incidents seem to comprise a tiny portion of the overall microblog environment. Most Chinese microbloggers are found to primarily share and forward jokes, images and videos, most of which are not original and are mostly duplicate others’ messages [13].

According to our literature review, much previous research on microblogger’s behavior, both Chinese and international studies, was conducted by using non-random sampling from the microblog population. For instance, microblog post samples were collected preferentially from active users (via public timeline API offered by Twitter) [14], [15] or sampled from popular topics posted on the web [13]. If not involving actual bias, those samples may incompletely reflect the real picture of the microblogging universe where the majority may be light users and where in any case evidence suggests a huge gap in frequency of usage. Previous study has used random digital search method for sampling Chinese blogs [16]. But no study has attempted to sample the Chinese microblogger space using a systematic approach and the probability sampling technique, in which each user has equal probability to be chosen into the sample, which is a pillar of many social science studies. Self-reported telephone survey was common approach to collect media use data in the past but the validity of this methodology is weakened by low response rate and selection bias [17]. As there is no representative sample for study, the real picture of how Chinese Internet users are using microblog services is unclear. To fill this gap, this study deploys random sampling techniques to explore a representative picture of microblogging behavior in China.

Thus, we come to our first and the second research questions, which are aimed at examining the characteristics of Chinese microbloggers from a representative sample.

Research Question 1: What are the demographic and geographical characteristics of Chinese microbloggers?

Research Question 2: What are the usage patterns of Chinese microbloggers?

We also investigate the factors that are able to predict the use of microblogging in China. Such research is only available concerning Twitter, but it does not yet explore Chinese microbloggers. For example, previous research on Twitter has found the number of users’ “tweets” is linked to number of followers of a specific microblogger and to number of a user’s “friends” (that is, microbloggers whom a user follows) [18]. Another study finds that number of friends is a better predictor for a user’s tweet frequency than number of followers [19]. Moreover, a user’s retweeting count is a unique dimension to rank user’s popularity and is not strongly correlated with number of followers and page rank score [18]. Another study reveals that number of followers or friends, age of the account since it was created, and the presences of weblinks or hashtags are all significant predictors for the rate of retweeting [14]. With such background, we have two more research questions.

Research Question 3: What are the predictors for a Chinese microblogger’s posting and reposting behaviors?

Research Question 4: What are the predictors for a Chinese microblogger’s rate of being reposted and receiving comment by other microbloggers?

## Methods

### Ethics Statement

The study was approved by the Human Research Ethics Committee for Non-Clinical Faculties, The University of Hong Kong. Data were obtained from Sina Weibo’s API (Application Programming Interface, http://open.weibo.com/). Before data collection, a developer account was granted by Sina Weibo to the Principal Investigator of this study, which allows the access to the data. Indirect identifier data fields, for example user identify code or user display, will be replaced to unidentifiable pseudo code after all data are collected upon the end of the project.

### Data Collection

Using a combination of computer programs on Linux and scripts that query the Sina Weibo API, we obtained a random sample of Sina Weibo user accounts and their latest posts, when available. All valid user accounts on Sina Weibo had an identity code of 10 digits. We also found the approximate maximum number based on our account database obtained by regular data collection for another project [20]. Using this approach, we generated randomly possible account codes between 1000000000 and the maximum (in this case, it was 4294917290) and successively tested their validity with the computer program on the Sina Weibo website. For example, for a randomly generated identity code 1801443400, we issued a Linux shell command “curl –I http://weibo.com/1801443400”.

Different responses were returned. Most often, we got a “/sorry?usernotexists” value for the “Location” HTTP header, which means that the user identity code did not exist. Other “Location” header values were considered to be potential hits, including redirections to signup or redirections to a permanent short URL with the users’ username. Once the code was confirmed to be valid, we deployed Sina Weibo API to obtain the user information, including gender, location, follower count, friend count, and the date of opening account.

We fetched information from the Sina Weibo API and stored the data in our database. When a valid user was found, its code was appended to a separate text file and its information was saved in the database. If the API returned an error, we ignored it. In those cases, we assumed those were users recently deleted, for spam or other reasons, which may explain why the website access still gave a potential hit.

After obtaining the list, we used another API call to fetch the sample’s user timeline. The Sina Weibo allows the API call to get the latest 200 posts of a given user and does not allow user setting to block outsider’s access.

Statistics for the sample we collected between January 18 and 23, 2012 are as follows: we generated 365,967 codes randomly, of which 335,941 were invalid and 28 obtained API error. Then, 29,998 valid account codes were obtained. The proportion of valid account code was 8.20% (30,026/365,967, 95% confidence interval CI 8.11%,8.29%). Assuming that the microblog account population is 3.29 billion (4294917290 minus 1000000000 plus 1), the total number of valid account was projected to be 269.7 million (95% CI 266.8 million to 272.6 million).

Weibos posted by the sampled microbloggers in the last 7 days before data collection were analyzed in this study. In total, we collected 21,030 posts (tweets, in Twitter parlance), of which 8,139 were original posts (non-retweets) and 12,891 were reposts (retweets) that constituted 12,127 unique identifiable code messages. The extent to which the samples’ written messages were reposted and commented on by other microbloggers was operationalized by using the maximum repost count and the maximum comment count among all the posts within the seven-day period. The counts were obtained via API calls. All these information was used to address Research Question 1 and 2.

To address Research Question 3 and 4, regression models were developed to predict the samples’ counts of original posts, unique reposts (the posts with unique identity code that were reposted by a specific user), maximum count of being reposted (the posts created by a specific user that were reposted by other microbloggers) and maximum count of receiving comments. Independent variables consisted of follower count, friend count, gender, province (binary categories: major cities Beijing/Shanghai/Guangdong or not), and number of days since account created.

Over-dispersed and zero-inflated microblog count data were analyzed by using hurdle regression [21]. Hurdle model is a count-based regression technique especially useful for modeling data with exceeding zero. It models the count outcomes as two separate components - one determining whether it is zero or non-zero and one generating the positive non-zero values. In the hurdle models used in this study, the zero portion (binary outcome of whether a count variable is a zero or not) was modeled by a binomial probability model. If the value is non-zero, conditional distribution of the count portion was analyzed by a zero-truncated negative binominal model, which is appropriate for modeling over-dispersed data. R version 2.14.1 [22] and package pscl [23] were deployed to conduct the statistical analysis. Log-likelihood and AIC were deployed to measure the model’s goodness of fit. The observed and the model-predicted percentages of zero count were compared. Log-transformed counts of followers and friends were found to be better predictors in term of log-likelihood statistics and were therefore used in the models.

## Results

Of the 29,998 collected samples’ statuses, 17,224 (57.4%, 95% CI 56.9%, 58.0%) were empty, i.e. no post was found in their timeline. When compared to the other 12,774 non-zero statuses users, the 17,224 samples had higher proportion of self-reported male (63.5%, χ^2^(1) = 150.7, p<0.05), considerably lower counts of followers (median = 0, Wilcoxon rank sum test, p<0.05) and friend count (median = 1, Wilcoxon rank sum test, p<0.05), and were more likely to report residential location as “others” (15.7%, χ^2^(1) = 128.8, p<0.05).

**Figure 1:**
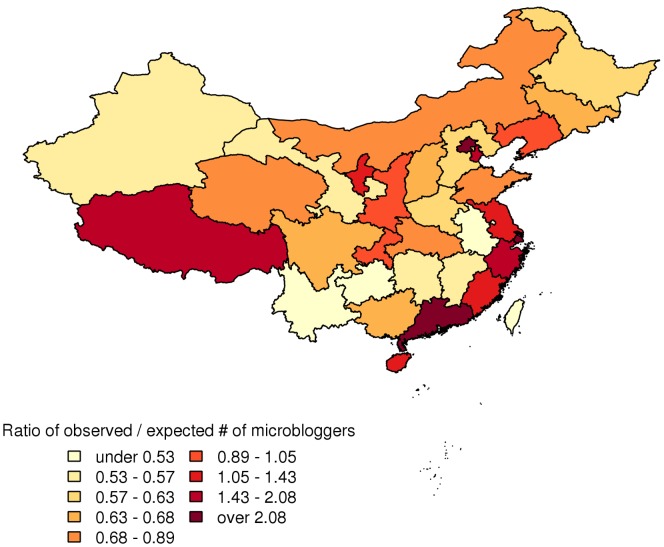
Geographical distribution of the sample.

In the following analysis, we examine the characteristics of the 12,774 samples with non-zero statuses count. The data shows that 56.5% of them (95% CI 55.6%,57.4%) were self-reported as males and 43.5% (95% CI 42.6%,44.4%) claimed themselves to be female. The top three self-reported locations where they reside were Guangdong (15.7%, 95% CI 15.1%,16.3%), Beijing (7.7%, 95% CI 7.2%,8.2%), and Jiangsu (5.3%, 95% CI 4.9%, 5.7%). International users constituted 1.6% (95% CI 1.3%,1.8%). When adjusting for variations in provincial population, observed-to-expected ratio (O/E ratio), i.e. expected proportion is the ratio of provincial population over the total Chinese population, of each location is computed and presented in Figure 1. Beijing (6.12), Shanghai (3.17), and Guangdong (2.35) were among the top O/E ratio locations. Other top ranked places included Macau (6.65), Hong Kong (1.94), and Tibet (1.79).

**Figure 2:**
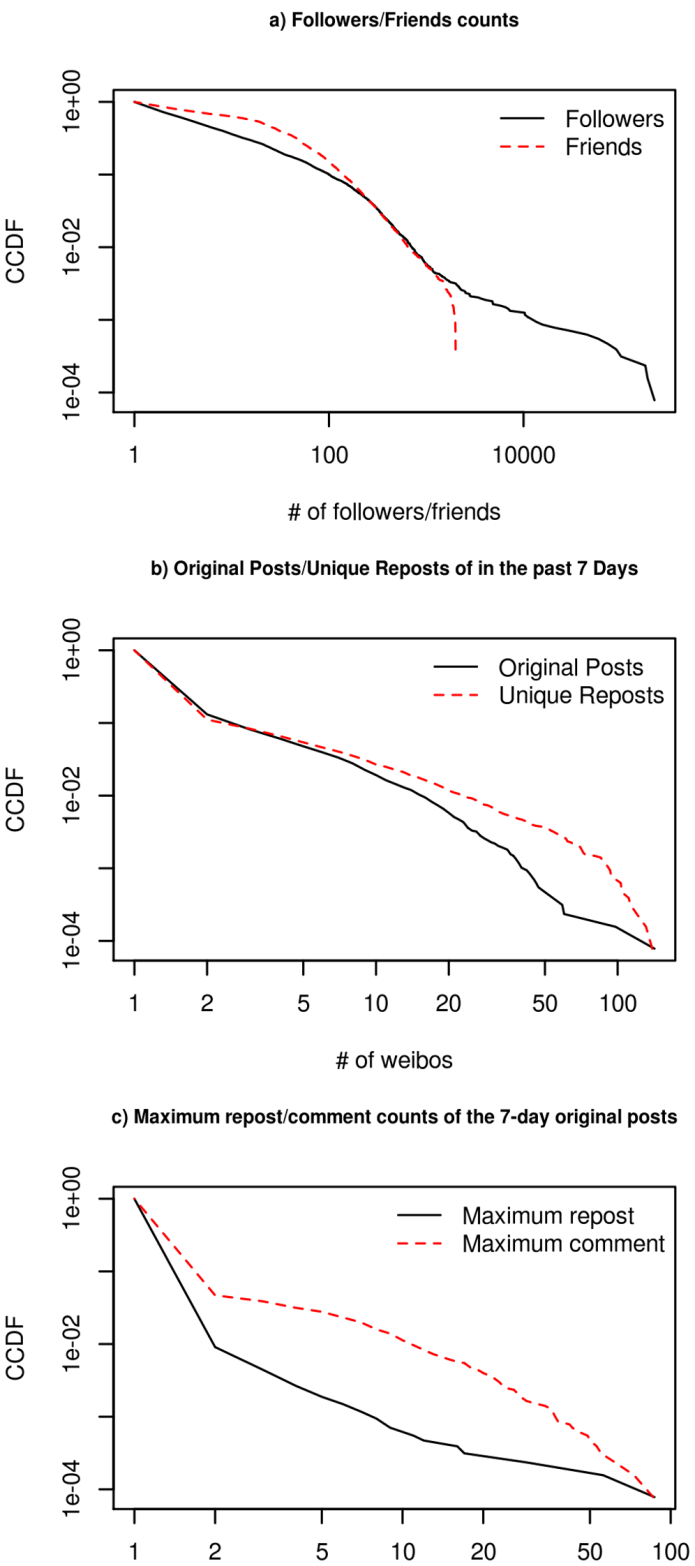
Usage characteristics of the sample.

As shown in Figure 2a, the distributions of the samples’ follower and friend counts were highly skewed and power-law distributed where the alpha exponents were 1.38 and 1.21 respectively. The y-axis of all charts in Figure 2 represents complementary cumulative distribution function (CCDF), which is chosen for comparison to previous Twitter research [18]. The medians of the samples’ follower and friend counts were 3 and 20 respectively.

The statistical characteristics of the weibo usage of the samples are as follows: total count of statuses (mean = 108.0; standard deviation = 495.6; the same as below), microblogs posted in the past seven days (1.64; 8.18), original posts in the past seven days (0.64; 3.23), and unique reposts in the past seven days (0.95; 5.69). Their distributions are shown in Figure 2b. When considering the number of posts within the past seven-day, 86.9% (95% CI 86.2%,87.4%) contributed no original posts, whereas 0.51% (95% CI 0.4%,0.65%) wrote 20 or more posts. We found that 88.9% (95% CI 88.3%,89.4%) did not repost any other’s post but 0.45% (95% CI 0.35%,0.60%) reposted more than 40 unique messages written by others in the past seven days.

All 21,030 collected posts within the seven-day period were created by 2,156 microbloggers (16.9%, 95% CI 16.2%,17.5%). Among all posts, 8,139 original posts were made by 1,677 microbloggers (13.1% 95% CI 12.5%,13.7%). There were 479 microbloggers (3.7%, 95% CI 3.4%,4.1%) who only reposted other’s messages but made no original post, whereas 735 microbloggers (5.8%, 95% CI 5.4%,6.2%) only contributed original weibos and made no repost. Those who posted 14 or more weibos within seven days (18.4% of 2,156) comprised 67.7% (95% CI 67.1%,68.3%) of all posts and those who posted eight or more original weibos within seven days (17.2% of 1,677) constituted 58.5% (95% CI 57.4%,59.6%) of all original posts.

Among the 8,139 original weibos, the means and standard deviations of the counts of being reposted and receiving comment were: being reposted (mean = 0.16; standard deviation = 1.90; the same as below) and receiving comments (1.46; 4.23). Figure 2c shows their distributions. Only 202 original weibos were reposted at least one time, whereas 15 of them were reposted at least 10 times. Only 117 microbloggers’ (0.9%, 95% CI 0.75%,1.08%) original contributions were reposted at least one time. On the other hand, 4,671 weibos received no comment but 69 of them received comments over 20 times. The original weibos that were attached with at least one comment were made by 599 microbloggers (4.69% 95% CI 4.33%,5.07%).

The sample’s original post count and unique repost count in the past seven days were modeled by hurdle regression as shown in Table 1. The theta parameters of both models were found to be statistically significant, suggesting over-dispersion and supporting the choice of hurdle modeling. In the count portion, original post count was positively associated with the number of followers (p<0.001). In the zero portion, samples with the following factors had higher likelihood to have non-zero original post count: being females, residing in major Chinese cities (Beijing/Shanghai/Guangdong), and smaller number of days since the account was created. We found that 83.7% of the samples were predicted by the model to make no original post, against the observed 86.9%.

**Table 1:** Predicting counts of original posts and unique reposts of the sample by using the hurdle model (n = 12,774).

	Original Posts			Unique Reposts		
	Estimate(SE)	z	p	Estimate(SE)	z	p
Count portion						
Intercept	0.18(0.35)	0.5		−0.94(0.41)	−2.3	*
Log count of followers	0.56(0.04)	12.4	***	0.27(0.05)	5.35	***
Log count of friends	−0.06(0.05)	−1.42		0.36(0.06)	6.09	***
Gender	−0.06(0.09)	−0.66		−0.31(0.1)	−3.04	**
Province (Beijing/Shanghai/Guangdong)	0.12(0.09)	1.35		0.16(0.1)	1.61	
Log days since created	−0.34(0.05)	−6.59	***	−0.08(0.07)	−1.21	
Log(theta)	−1.9(0.29)	−6.65	***	−1.84(0.22)	−8.22	***
Zero portion						
Intercept	0.1(0.21)	0.5	***	−2.08(0.25)	−8.31	***
Number of Followers	0.68(0.03)	27.14	***	0.7(0.03)	25.29	***
Number of Friends	−0.05(0.03)	−1.98	***	0.2(0.03)	6.16	***
Gender	−0.29(0.06)	−4.94	***	−0.54(0.07)	−8.01	***
Province (Beijing/Shanghai/Guangdong)	0.43(0.06)	7.08	***	0.09(0.07)	1.23	
Log days since created	−0.61(0.03)	−17.58	***	−0.36(0.04)	−8.72	***
Log-likelihood	−7708 (DF = 13)			−7156 (DF = 13)		
AIC	15441.2			14338.5		
Observed % of zero	86.9%			88.9%		
Predicted % of zero	83.7%			80.5%		

* p<0.05, **p<0.01, ***p<0.001.

Remark: SE stands for standard error; DF stands for degree of freedom.

Unique repost count was positively linked to both follower count (p<0.001), friend count (p<0.001), and being female (p<0.01). Those having larger numbers of followers and friends, being female, or smaller number of days since the account was created were more likely to have non-zero unique reposts. 80.5% of the samples were predicted by the model to post zero unique reposts, when compared to the observed 88.9%.

Table 2 presents the models predicting the sample’s maximum counts of being reposted and comment received. The theta parameters of both models were found to be statistically non-significant, showing no evidence of over-dispersion. Microblogs posted by those who have a larger follower count (p<0.001) or smaller number of days since the account was created (p<0.05) were more likely to be reposted by other microbloggers. Fewer followers and friends were associated with a larger chance to have a null count. The model predicted 99.4% of zero count, and the observed percentage was 99.1%.

**Table 2:** Predicting the sample’s maximum counts of being reposted and commented by using hurdle model (n = 12,774).

	Count of being reposted (maximum)			Count of receiving comments (maximum)		
	Estimate(SE)	Z	p	Estimate(SE)	z	p
Count portion						
Intercept	−0.47(4.78)	−0.1		−0.82(0.43)	−1.91	
Log count of followers	0.78(0.19)	4.18	***	0.4(0.06)	6.38	***
Log count of friends	−0.46(0.26)	−1.75		−0.06(0.08)	−0.76	
Gender	0.31(0.4)	0.78		0.22(0.1)	2.13	*
Province (Beijing/Shanghai/Guangdong)	0.12(0.38)	0.31		0.09(0.1)	0.93	
Log days since created	−0.66(0.31)	−2.13	*	0.08(0.07)	1.11	
Log (theta)	−3.21(4.84)	−0.66		−0.2(0.12)	−1.65	
Zero portion						
Intercept	−8.49(0.93)	−9.12	***	−5.43(0.42)	−13.02	***
Number of Followers	0.65(0.06)	10.15	***	0.75(0.04)	18.93	***
Number of Friends	0.28(0.09)	3.06	**	0.12(0.05)	2.52	*
Gender	−0.05(0.2)	−0.23		−0.45(0.1)	−4.69	***
Province (Beijing/Shanghai/Guangdong)	0.36(0.2)	1.83		0.33(0.1)	3.42	***
Log days since created	0.05(0.15)	0.32		0.01(0.07)	0.21	
Log-likelihood	−683 (DF = 13)			−3364 (DF = 13)		
AIC	1392.0			6754.3		
Observed % of zero	99.1%			95.3%		
Predicted % of zero	99.4%			86.4%		

* p<0.05, **p<0.01, ***p<0.001.

Remark: SE stands for standard error; DF stands for degree of freedom.

Having more followers (p<0.001) and being male (p<0.05) were found to garner larger amount of comments from their posts. Those having larger numbers of followers and friends, being female or residing in major Chinese cities were more likely to have a non-zero count of comment. The model predicted 86.4% with zero comment count, and the observed figure in the sample was 95.3%.

## Discussion

### Characteristics of Chinese Microbloggers

Based on self-reported data, the gender distribution of the Chinese microbloggers is found to be predominantly male: 56.5% males and 43.5% females respectively. It seems to suggest a larger portion of male microbloggers in China. But we should be at first cautious to the self-reported data that may be not completely reliable. However, as the gender distribution of the sample is almost the same as the one obtained from survey method [3], the sample data appear to reflect the overall Chinese microblog landscape. Such gender distribution is contrary to the global Twitter users that are female preponderance [24]. It may be attributable to cultural difference in technology use between East and West. Another possible reason may be that Chinese microblog platforms provide not only social network service, but also integration with online discussion forum functions that are usually male in majority.

In the sample, more than one fourth of the Chinese microbloggers reported their residence as in major cities such as Guangdong (15.7%), Beijing (7.68%), and Shanghai (4.67%). These three places have disproportionately higher ratio of microbloggers in the population. Based on overall Internet user distribution data in China [1], these three cities account for only 9% of the total Chinese Internet population, indicating their over-representation in the microblog domain. While a geographical imbalance in Internet use has been observed in China [25], the discrepancy in Internet use was found to be more pronounced for microblogging.

Distributions of follower and friend counts are markedly positively skewed, such that three fourths of Chinese microbloggers had less than 20 followers and the half of them had less than 20 friends, which is not surprising as a similar power-law distribution has been identified in the Twitter research [18].

### Pattern of Microblog Use

A number of interesting patterns of microblog use in China are observed. First, a significant portion of accounts are inactive in term of microblog contribution. In the 29,998 samples, about 57.4% of their statuses were empty. Among the non-empty statuses microbloggers, more than half had less than 5 weibos in their statuses. It is plausible that many Chinese Internet users seem to just surf the microblog but do not often produce contents, which is very similar to what has been found on Twitter [26]. Moreover, a lot of Chinese microblog users may first sign up with the service but then seldom make use of it. It would also be attributable to the provider’s cross-platform promotion (Sina is one of the leading email and portal service provider in China) and aggressive marketing campaign that as a result has attracted a large group of idle users. Another possible reason is that some of these accounts could be the so-called “zombie” accounts which have been created only to become friends or followers of other accounts. Thus these accounts are not active in posting or reposting statuses. In any case, because subscribers number is often used for evaluating company value of a social media enterprise, such data are important to the understanding of the microblog industry in China.

Another observation is that a significant amount of original posts and reposts were created by a small group of microbloggers. Only 13% (95% CI 12.6%,13.7%) of the Chinese microbloggers made at least one original post in a seven-day period. An even smaller fraction of microbloggers, about 4.8% (95% CI 4.4%,5.2%), contribute a disproportional amount of original posts, more than 80% (95% CI,78.6%,80.3%) of the totals. In terms of information diffusion, there was only a tiny group of users, about 4.8% (95% CI 4.5%,5.2%), whose microblogs were reposted or received comments at least once. This finding evidences that a small active group appears to draw most of the microbloggers’ attention and seems to dominate the overall microblog space. It also poses a challenge to the common sampling approach used in previous studies [13], [14], [15], in which posts or tweets were sampled from a public timeline and the hot topics. Thus the sample is markedly biased toward a small number of active microbloggers and is not representative of the overall microblog space.

### Predictors of Posting and Reposting

Previous studies did not often classify posts or tweets into contributing original posts and reposting other’s messages [18], [19]. However, these two types of microblogging behaviors are motivated differently, and thus the previous findings would be misleading. In this study, we disentangle them and the two variables are separately regressed on two different models. Our findings reveal follower count is a better predictor than friend count to determine frequency of posting original weibos. Moreover, we found that users with larger follower and friend pools are more likely to create reposts, which reinforces our assumption that creating original posts and reposting other messages are two distinct behaviors of using microblog. Creating original posts is solely determined by the quantity of your audiences, i.e. follower count – the larger the audience, the more actively the blogger contributes original pieces to audiences. Whereas, reposting other’s messages is linked with quantity of audience as well as quantity of content creators a user is following, i.e. friend count. One possible explanation is that when a microblogger has more followers, the microblogger expects that a post would have a higher chance to be read by more people and would have a stronger incentive to write. The more a user reads other people’s posts, the more a microblogger seems to be more motivated to redistribute the post. Drawing on the theory of uses and gratifications and the conclusions we obtain from the findings, this study may direct us to theorize that microblogging behaviour is a need- and-gratification-based activity in which people use microblogs to gratify specific communication needs and keep connected with their social circles [27].

Regarding the individual power of being reposted and receiving comments, follower count is still a key determinant in getting the weibo message across. This result would be less surprising under the assumption that the larger the group of people who are reading an individual’s microblog contributions, the easier it becomes to have one’s microblog reach a widespread audience. But this ability is unrelated to the quantity of microbloggers the person is following. Moreover, it is interesting to observe that gender plays a role in shaping comment count. When the follower count and friend count are factored in, a male microblogger’s post is apt to drive more comments than is the case with a female user if the comment count is non-zero. But at the same time, male users are more likely to receive zero comments.

The above findings are useful in various social media applications. For example in business application, if a company wants to spread a marketing message through a microblog, it will be possible to identify the potential microbloggers who are most likely to repost and spread out the message based on our analysis.

### Conclusions

In this paper we report our exploratory study of a set of randomly sampled microblogs in China. Our study has revealed the characteristics of the microbloggers and some of their behaviors. We believe that these data provide an unbiased picture of the Chinese microblog sphere and can be used as the baseline reference for future research in this area.

As the current study is exploratory, it will be a good future research direction to further investigate and reconfirm the statistically significant relationships that we identified in this study. It will be interesting to study the theoretical foundation behind the relationships between number of posts, reposts, and other factors.

As shown in our results, there are a large number of accounts with very few or even no posts. We suspect that some of these accounts are undetected “zombie” accounts. However, it is not easy to distinguish the difference between such accounts and an authentic account with no activities. One possible future research direction is to investigate how various techniques such as data mining can be used to classify these accounts.
